# Idiopathic Harlequin Syndrome Manifesting during Exercise: A Case Report and Review of the Literature

**DOI:** 10.1155/2017/5342593

**Published:** 2017-02-21

**Authors:** Hussein Algahtani, Bader Shirah, Raghad Algahtani, Abdulah Alkahtani

**Affiliations:** ^1^King Abdulaziz Medical City, King Saud bin Abdulaziz University for Health Sciences, Jeddah, Saudi Arabia; ^2^King Abdullah International Medical Research Center, King Saud bin Abdulaziz University for Health Sciences, Jeddah, Saudi Arabia; ^3^King Saud bin Abdulaziz University for Health Sciences, Jeddah, Saudi Arabia

## Abstract

Harlequin syndrome is a rare autonomic disorder characterized by unilateral facial flushing and sweating with contralateral anhidrosis induced by exercise, heat, and emotion. It is usually idiopathic but could be the first manifestation of several serious underlying medical conditions. Medical or surgical treatments are not required for idiopathic Harlequin syndrome, but social and psychological factors may indicate sympathectomy or botulinum toxin injection. In this article, we report a case of idiopathic Harlequin syndrome and review the literature.

## 1. Introduction

Harlequin syndrome is a rare autonomic disorder characterized by unilateral facial flushing and sweating with contralateral anhidrosis induced by exercise, heat, and emotion [1]. In this article, we report a case of idiopathic Harlequin syndrome and review the literature.

## 2. Case Report

A 35-year-old female presented with a 6-month history of right side hemifacial flushing and sweating during exertion. This occurred especially when she exercised more than an hour. There was no history of trauma, smoking, cough, loss of appetite or weight, or exposure to any procedure or surgery. Her past, family, and social history were unremarkable. Clinical examination at rest showed normal vital signs and systemic examination, including skin. Her neurological examinations including higher mental function, cranial nerves, motor and sensory systems, deep tendon reflexes, and coordination were all normal. Specifically, there were no pupillary abnormalities, including Horner syndrome. The patient was instructed to exercise for more than an hour and report immediately to the clinic for physical examination. Examination immediately after exercise revealed unilateral red discoloration of the face and upper neck with sweating. Magnetic resonance imaging (MRI) of the brain and cervicothoracic spine was normal. Computerized tomography (CT) scan of the chest, including the area of the thoracic sympathetic chain, was normal. Since the workup and imaging were completely unremarkable, a diagnosis of Harlequin syndrome was made, and she was reassured with no treatment given since she was not bothered by her symptoms. The patient was followed up in the clinic for several years with persistence of symptoms and normal health status.

## 3. Discussion

Harlequin is a fictional character of European folklore with a red and black face mask [2]. Harlequin syndrome was first described by Lance et al. [3] in 1988 when they investigated five patients who complained of sudden onset of unilateral facial flushing and sweating during exercise or in hot conditions. They proposed that the torsional occlusion of the anterior radicular artery at the third thoracic segment caused the syndrome. In 1993, they described two further patients plus two from the original report, and they concluded that the lesion might involve both the pre- and postganglionic cervical sympathetic fibers and parasympathetic neurons of the ciliary ganglion [4]. It is now known that hemifacial cutaneous sympathetic denervation is the cause of this rare autonomic disorder [2].

**Figure 1:**
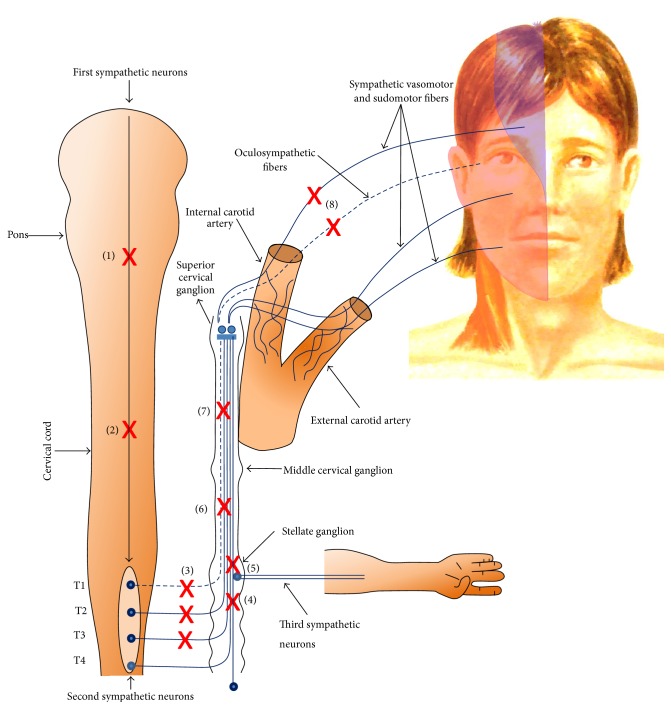
Diagram demonstrating the sympathetic innervation to the face with possible lesion sites. (1) = lesions within the pons, (2) = lesions within the spinal cord, (3) = lesions within the thoracic roots T1–T3, (4) = sympathetic chain between T1 and T2, (5) = stellate ganglion, (6) = sympathetic chain between stellate and middle cervical ganglion, (7) = sympathetic chain between middle and superior cervical ganglion, and (8) = sympathetic fibers traveling with the internal carotid artery.

The face is innervated by the sympathetic fibers that are originated from the hypothalamus (first-order neuron) and synapsed in the lateral horn of the spinal cord with the preganglionic (second-order) neurons. The vasomotor and the sudomotor fibers exit the spinal cord at T_2-3_ and pass through the sympathetic chain in order to synapse in the superior cervical ganglion. The postganglionic fibers (third-order neuron) then proceed inside the carotid plexus to arrive at their effectors. A lesion in the ipsilateral sympathetic fibers may lead to loss of flushing and anhidrosis on the same side of the face associated with contralateral excessive flushing and sweating. Harlequin syndrome is caused by a unilateral blockage of the sympathetic innervation of the face which results in inability of the facial vasculature to dilate in response to normal stimuli. The result is usually unilateral lack of flushing and anhidrosis [2] (Figure 1).

Harlequin syndrome is usually idiopathic but could be the first manifestation of several disorders such as Guillain-Barré syndrome, Bradbury-Eggleston syndrome, and diabetic neuropathy. The syndrome also might be caused by brainstem infarction, carotid artery dissection, toxic goiter, superior mediastinal neurinoma, syringomyelia, multiple sclerosis, internal jugular vein catheterization, and iatrogenic effects of invasive procedures [5]. It is usually acquired but can be congenital in up to 6% of the reported cases [6].

Harlequin syndrome and Horner syndrome share some similarities but also have some differences. Unilateral facial flushing is the shared feature, but ocular phenomena including ptosis and miosis are present only in Horner syndrome. Detailed clinical examination, testing of autonomic functions, and appropriate imaging techniques may help to describe additional abnormalities and localize the site of sympathetic deficit. Clinical examination in suspected cases should particularly include a search for pathology in the area of the thoracic sympathetic outflow and assessment of pupillary responses and deep tendon reflexes [7].

The radiological findings in Harlequin syndrome are typically normal. It is important to perform CT and MRI of the brain and cervicothoracic spine with the area of the thoracic sympathetic chain to exclude space-occupying lesions, infarction, or other etiologies [8]. Other important examinations that can be done in order to understand the complex differential diagnosis of this syndrome include total sweating test to recognize areas of anhidrosis, cardiovascular reflex tests to recognize the presence of autonomic failure, microneurography from the peroneal nerve to record muscle sympathetic nerve activity and skin sympathetic nerve activity in the same innervation field, and a skin biopsy. Such tests are usually done in centers that are specialized in autonomic nervous system disorders.

Harlequin syndrome typically does not require medical or surgical treatment. Surgical sympathectomy ipsilateral to the affected side may be performed to prevent compensatory flushing and sweating. However, this procedure is not always successful and can be complicated by further compensatory flushing and sweating in another location. Repeated stellate ganglion blocks might be used either as a preoperative screen for sympathectomy or as a less invasive alternative treatment [9]. Botulinum toxin was recently reported as effective, safe, and minimally invasive with a high degree of satisfaction [10].

## 4. Conclusion

Harlequin syndrome is a rare autonomic disorder characterized by unilateral facial flushing and sweating with contralateral anhidrosis induced by exercise, heat, and emotion. It is usually idiopathic but could be the first manifestation of several serious underlying medical conditions. Medical or surgical treatments are not required for idiopathic Harlequin syndrome, but social and psychological factors may indicate sympathectomy or botulinum toxin injection.
